# Evidence for Unintentional Emotional Contagion Beyond Dyads

**DOI:** 10.1371/journal.pone.0067371

**Published:** 2013-06-28

**Authors:** Guillaume Dezecache, Laurence Conty, Michele Chadwick, Leonor Philip, Robert Soussignan, Dan Sperber, Julie Grèzes

**Affiliations:** 1 Laboratoire de Neurosciences Cognitives (LNC), INSERM U960, and Institut d’Etudes de la Cognition (IEC), Ecole Normale Supérieure (ENS), Paris, France; 2 Institut Jean Nicod (IJN), UMR 8129 CNRS and Institut d’Etudes de la Cognition (IEC), Ecole Normale Supérieure, and Ecole des Hautes Etudes en Sciences Sociales (ENS-EHESS), Paris, France; 3 Laboratoire de Psychopathologie et Neuropsychologie (LPN, EA2027), Université Paris 8, Saint-Denis, France; 4 Centre des Sciences du Goût et de l’Alimentation (CSGA), UMR 6265 CNRS, 1324 INRA, Université de Bourgogne, Dijon, France; 5 Department of Cognitive Science, Central European University (CEU), Budapest, Hungary; Royal Holloway, University of London, United Kingdom

## Abstract

Little is known about the spread of emotions beyond dyads. Yet, it is of importance for explaining the emergence of crowd behaviors. Here, we experimentally addressed whether emotional homogeneity within a crowd might result from a cascade of local emotional transmissions where the perception of another’s emotional expression produces, in the observer's face and body, sufficient information to allow for the transmission of the emotion to a third party. We reproduced a minimal element of a crowd situation and recorded the facial electromyographic activity and the skin conductance response of an individual C observing the face of an individual B watching an individual A displaying either joy or fear full body expressions. Critically, individual B did not know that she was being watched. We show that emotions of joy and fear displayed by A were spontaneously transmitted to C through B, even when the emotional information available in B’s faces could not be explicitly recognized. These findings demonstrate that one is tuned to react to others’ emotional signals and to unintentionally produce subtle but sufficient emotional cues to induce emotional states in others. This phenomenon could be the mark of a spontaneous cooperative behavior whose function is to communicate survival-value information to conspecifics.

## Introduction

Emotional crowds - where groups of individuals come to adopt similar affective states and patterns of behavior through local interactions and without any prior centralized coordination [1] - were a major topic in the nascent field of social psychology in the Nineteenth and early Twentieth centuries. Social scientists such as Gabriel Tarde [2], Scipio Sighele [3] or Gustave Le Bon [4] theorized about the emergence of such collective behaviors and the psychological impact crowds have over their members. Crowds were characterized as *milieux* where affects spread very rapidly and in an uncontrollable manner (e.g., [4]). As a consequence, a group of individuals who are not acquainted with one another may spontaneously come to adopt the same behavior (e.g., a collective flight in crowd panic), giving the impression of 'mental unity' within the group [4]. A necessary condition for the emergence of such collective behavior is the propagation of emotions across individuals. How can emotional information circulate from one individual to another in a way that rapidly achieves emotional unity of the crowd?

Despite a few notable exceptions [5], [6], emotional transmission processes occurring in groups has been neglected by later social psychologists, probably due in part to the difficulty of producing group-like phenomena in laboratory settings [7]. Emotional contagion (here, the "tendency to automatically mimic and synchronize facial expressions, vocalizations, postures, and movements with those of another person and consequently to converge emotionally" [8]) is commonly studied in dyadic interactions (see [8] for an extensive review). If however emotional homogeneity within a crowd is to be achieved through transmission from individual to individual, it is not sufficient that humans should be tuned to catch others' emotions in dyadic interactions. It is also critical that humans should be tuned to reproduce the emotional cues they observe to a degree sufficient to spontaneously spread emotional information to other crowd members. This is needed for emotions to be *transitively contagious*: the perception of individual A's emotional expressions by individual B should ultimately affect the emotional experience of an individual C who is observing B but not A. Such a minimal situation of *transitive emotional transmission* may be, we surmise, at the basis of emotional contagion on a much larger scale. What is also critical here is that emotional contagion should take place automatically rather than as a result of people’s decision to influence others or to accept such influence.

**Figure 1:**
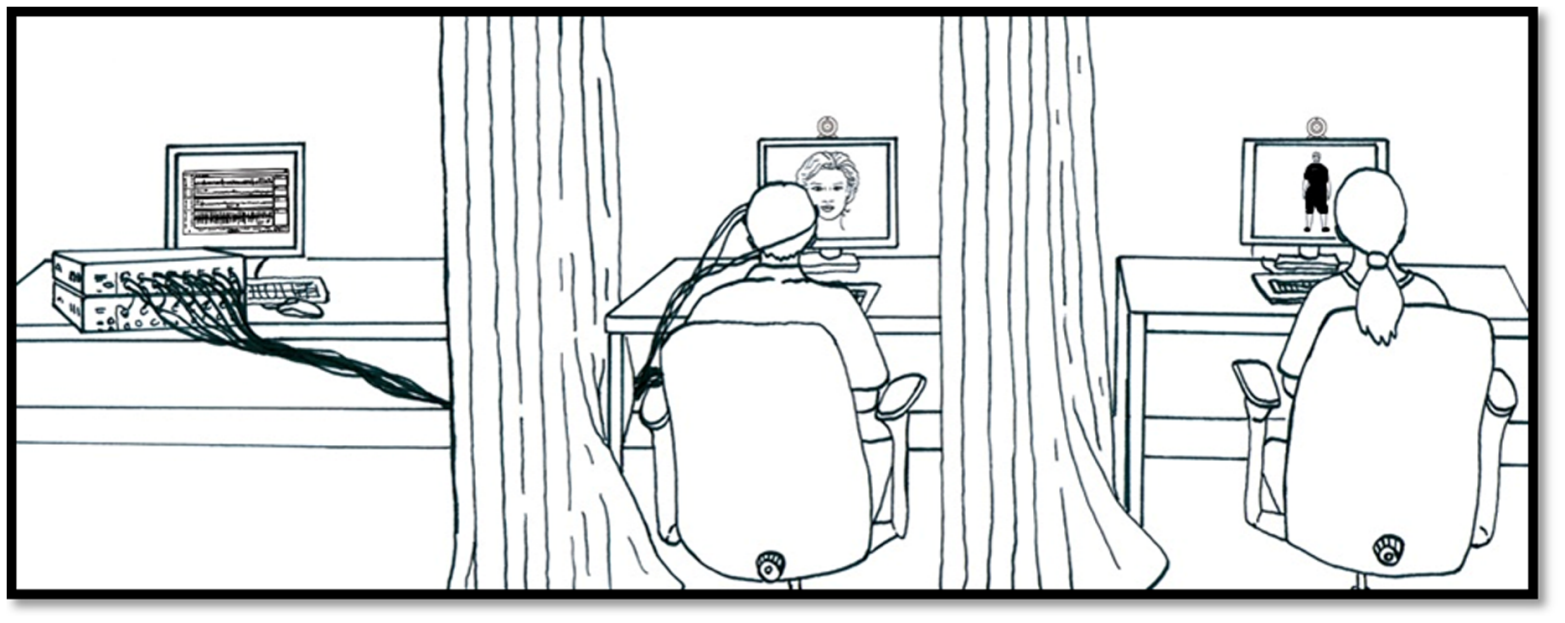
The experimental apparatus. Participant B (on the right of the picture) is isolated from participant C (in the middle) by means of a large black folding screen. On the left of the picture is the recording device, concealed to C. Stimuli were presented to B using a screen located in front of her; a webcam was placed on top of the screen so as to display B's face on C's screen.

In the present study, we investigated the transmission of emotional information in transitive triadic chains where the behavior of an individual A was observed by a participant B who was herself observed by a participant C. Joy and fear were chosen as target emotions because of their relevance to coordinated behavior and, arguably, their survival and fitness value [9], [10] makes it particularly likely that they should easily spread in groups. As both motor and affective processes are implicated in emotional contagion [11], [12], we recorded in a first experiment, the electromyographic (EMG) activity of *zygomaticus major* (ZM) and *corrugator supercilii* (CS), two muscles that are respectively involved in the production of smiling and frowning [13] and may be differentially induced by unseen facial and bodily gestures of joy and fear [14]. The activity of ZM and CS, as well as the skin conductance response (SCR) (a measure of physiological arousal [15]), were recorded in participants C while they were watching a participant B’s face. B herself was either watching a video of full bodily expressions including vocalizations of joy or fear displayed by another individual A, or, in a control condition, a video without social or emotional content. While our protocol was designed to investigate transitive emotional transmission in terms of facial patterns and physiological arousal in C, it was not designed to investigate the mechanisms behind facial reactions: whether these facial reactions qualify as rapid facial reactions [16] and whether they are mediated by motor-mimetic [17], [18] or affective/emotional appraisal [16], [19], [20] processes cannot be addressed here. As shown in figure 1, participants B and C were sitting in adjacent booths during the experiment. While numerous studies did report an impact of the presence of an audience on the intensity of facial expressions of emotions [21], [22], importantly here, participants B were not informed that the participant in the adjacent booth was watching them.

Finally, to help determine the degree to which the emotional expressions of B were explicitly perceptible, and hence the nature of emotional transmission from B to C, we presented in a follow-up experiment the video recordings of individuals B’s faces to naive judges who were asked to label the emotional expressions of B.

We predicted that an emotion displayed by individual A would be transmitted to individual C via individual B (figure 1) even though B was not aware that she was being watched, and even when her emotional reactions could not be explicitly identified by individual C. Testing the transitivity of emotional contagion processes in this way may not only provide insight concerning the spread of affects in groups and crowds; it may also shed light on what may be the nature and function of emotional signaling mechanisms from an evolutionary point of view.

## Materials and Methods

### (a) Ethics Statement

We obtained ethics approval from the local research ethics committees (CPP Ile de France III and Institut Mutualiste Montsouris) for the two experiments. All provided written informed consent according to institutional guidelines of the local research ethics committee.

(b) Experiment 1

#### (i) Participants

Thirty male participants (mean age 24.6 y ±0.73 SE, range 18–36 y) were recruited to represent C in the emotional transmission chain. We chose female participants to represent individuals B in the transmission chain because numerous studies suggest that women are facially more expressive than men (e.g., [23]). Sixty female participants (mean age 24 y ±0.48 SE, range 18–36 y) were thus recruited to represent B. All of the participants had normal or corrected-to-normal vision, were naive to the aim of the experiment and presented no neurological or psychiatric history. All provided written informed consent according to institutional guidelines of the local research ethics committee and were paid for their participation. All the participants were debriefed and thanked after their participation.

#### (ii) Stimuli

The stimuli presented to B (and standing for A) consisted of 45 videos (mean duration 6060±20 ms, range 6000–6400 ms) of size 620×576 pixels projected on a 19-inch black LCD screen. The videos of emotional conditions depicted 15 actors (8 females, 7 males) playing joy (n = 15) and fear (n = 15), using facial, bodily as well as vocal cues. These videos were extracted from sessions with professional actors from the Ecole Jacques-Lecoq, in Paris, France. The stimuli of the non-social condition (n* = *15) displayed fixed shots of landscapes that were shot in the French countryside.

All stimuli were validated in a forced-choice task where 15 participants (6 females, 8 males, mean age 22.5 y ±1.46 SE) were instructed to determine the emotional content of the video, selecting from among 7 possible choices (anger, disgust, joy, fear, surprise, sadness or none). The stimuli were correctly categorized: joy stimuli were labeled as depicting joy (93% of the responses selected the ‘joy’ label, contra 4% for the ‘sadness’ label, and less than 1% for the five other labels); fear stimuli were labeled as depicting fear (97% of the responses selected the ‘fear’ label, contra less than 1% of the responses for the six other labels); finally, non-social stimuli were labeled as not depicting any emotion (94% of the responses selected the ‘none’ label, contra 4% for the ‘joy’ label, and less than 1% for the five other labels).

#### (iii) Overall procedure

After their arrival, the first two participants (one female participant, representing B; and one male participant representing C) were told that they will take part in two distinct experiments and were escorted to two separated rooms. The second female participant B was called in one hour later so as to replace the former female participant.

#### (iv) Specific procedure for participant B

**Figure 2:**
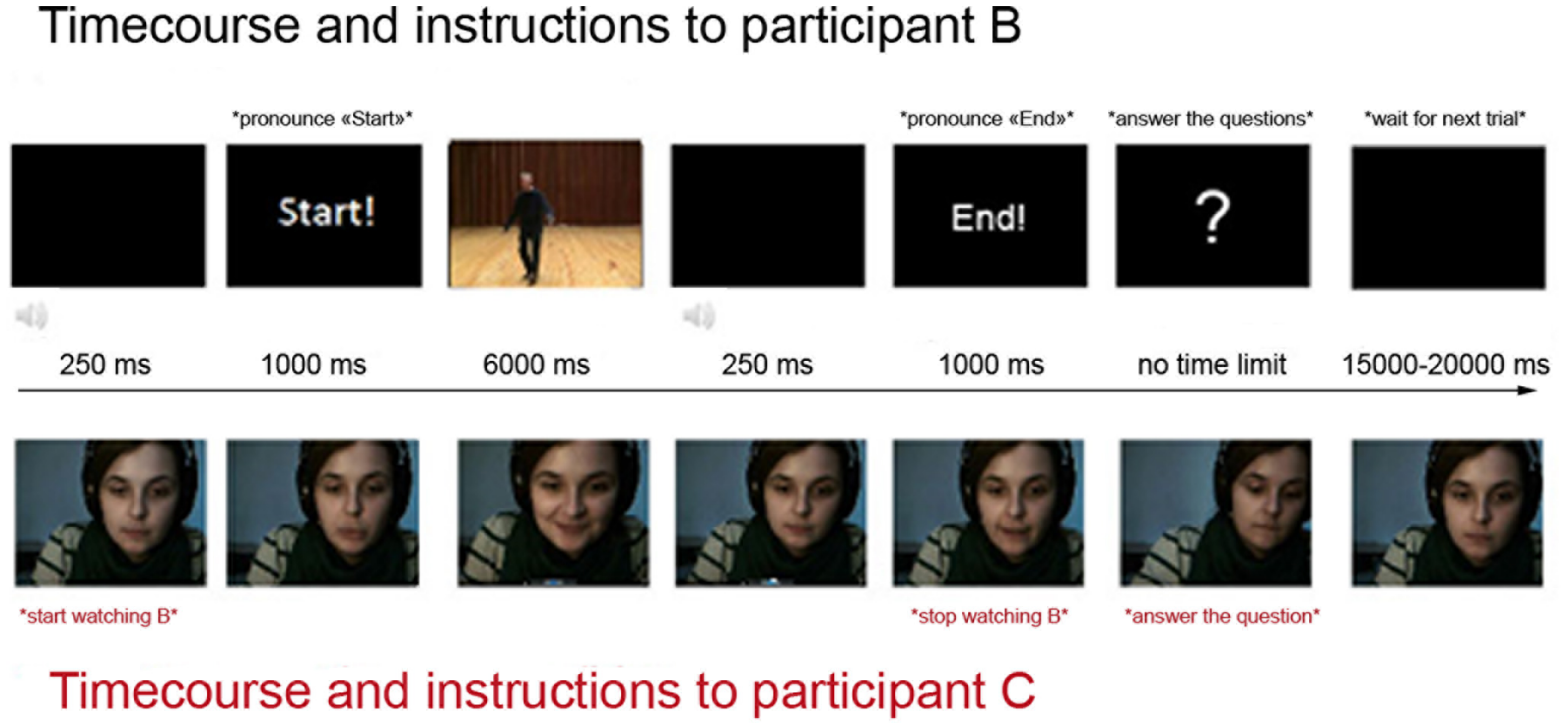
The experimental protocol timeline for participants B and C. Specific instructions are inserted between asterisks. The subject of the photograph has given written informed consent, as outlined in the PLOS consent form, to publication of her photograph.

While participant C was escorted to and set up in the experimental room, participant B underwent training in the experimental procedure in a waiting room, so as to lead B to believe that she was going to participate to a completely different experiment. The procedure (see figure 2) consisted in the presentation of the videos in a random order on a black LCD screen of size 19-inch. Each video was preceded by a 250 ms beep followed by the presentation of the word “Start” for 1000 ms. At the end of each video, the word “End” appeared on the screen for 1000 ms. B was instructed to pronounce these words sufficiently loudly to permit her speech to be recorded by the webcam’s microphone and was told that this would help the experimenter distinguish between the different trials in a further analysis. This was actually done to inform C that a video was beginning or ending. Furthermore, B was told that she would be filmed via a webcam placed on the top of the screen and that this was solely done to check whether she actually paid attention to the movies. In fact, her reflection was retransmitted onto C’s screen (figure 1) but none of our B participants reported being aware that she was being watched by another participant during the session. B was then asked to select the emotion displayed on the video in a forced-choice task, choosing the appropriate emotion from between three options (joy, fear, none) and to rate the intensity of the emotion on a 9-point scale. After having responded to these two questions, B waited for a period of time (between 15 and 20 sec) before a new video sequence began. Note that B was wearing headphones, and that this was done to improve the auditory input provided to B as well as to prevent any auditory cues about the content of the videos to be transmitted to C. Also, before joining participant C in the experimental room, Bs were told that there would already be somebody in the experimental room participating in an experiment led by another research team, and that it was important to enter the room as quietly as possible. At the same time, B was also told that the words “Start” and “End” would not disturb this other participant who was wearing headphones. Critically, during the experimental session, participant B never saw participant C who was hidden by a folding screen.

#### (v) Specific procedure for participant C

While participant B was instructed, participant C was installed in the experimental room (placing of EMG and SCR electrodes, see *(vi) Data acquisition*) and was told that he will watch two other participants (one after the other) watching different movies with non-social or emotional content. His task was to report on a sheet of paper, after each trial, what he thought the other participant had just seen between the words “Start” and “End”. C was also told to remain silent throughout the experiment.

#### (vi) Data acquisition

Using the acquisition system ADInstruments (ML870/Powerlab 8/30), we continuously recorded the EMG activity of C using Sensormedics 4 mm shielded Ag/AgCl miniature electrodes (Biopac Systems, Inc) (sample rate: 2 kHz; range: 20 mV; spatial resolution: 16 bits). Before attaching the electrodes, the target sites on the left of C’s face were cleaned with alcohol and gently rubbed to reduce inter-electrode impedance. Two pairs of electrodes filled with electrolyte gel were placed on the target sites: left ZM and left CS muscles [24]. The ground electrode was placed on the upper right forehead. Last, the signal was amplified, band-pass filtered online between 10–500 Hz, and then integrated. Integral values were then offline subsampled at 10 Hz resulting in the extraction of 100 ms time bins.

Concerning the recording of SCR, 2 bipolar finger electrodes (MLT116F) were attached with a Velcro™ attachment straps to the first phalanx of the index and middle-fingers of the non-dominant hand. The SCR was recorded at a sampling frequency of 2 kHz with a high-pass filter at 0.5 Hz, and then offline subsampled at 2 Hz resulting in the extraction of 500 ms time bins.

#### (vii) Data analysis

Due to the nature of our protocol (stimuli of long duration, expectation of signals of low amplitude), we deliberately chose not to prevent participant C’s free facial movements though they were instructed that they should not move their arms nor their head during the presentation of the stimulus. Consequently, we had to exclude those participants whose data were too noisy: data from ZM (n = 4 participants), CS (n = 8 participants) and SCR (n = 4 participants) were thus rejected prior to the analysis. Moreover, EMG trials containing artifacts were manually rejected, following a visual inspection. Participants with a high rate of trial rejection were excluded from the statistical analysis for the relevant signal, (n* = *3 for ZM, n* = *5 for CS), leaving a total of n = 23 for ZM, n* = *17 for CS for the statistical analysis. For SCR recordings, responses beginning before the first second following the video presentation were rejected and participants with a high rate of trial rejection or with absence of SCRs were excluded of the statistical analysis (n = 7) leaving a total of 19 participants for the statistical analysis.

Then, for EMG data, the pre-stimulus baseline was computed over 500 ms before the video onset. EMG activity per trial was obtained by extracting the maximal change from the baseline level occurring between 500 to 6000 ms after the video onset. As we could not predict when, in relation to B’s processing of the stimuli, C’s facial activity would occur, we considered the maximal activity in this large temporal time window.

For SCR data, the pre-stimulus baseline was computed over 1500 ms before the video onset. SCR activity per trial was obtained by extracting the maximal change from baseline level occurring between 1000 to 6500 ms after the video onset. Data for each trial was then natural-log transformed for both EMG and SCR activity.

Finally, data were submitted, separately for each physiological measure, to repeated measures ANOVA using Emotion (joy vs. non-social vs. fear) as within-subject factors. Taking into account the sphericity assumption, we adjusted the degrees of freedom using the Greenhouse-Geisser correction where appropriate (*ε* value). Finally, Bonferroni corrections were employed to account for multiple testing. Post-hoc comparisons were also performed for the analysis of simple main effects.

### (c) Experiment 2

#### (i) Participants

Three judges (2 female, 1 male, mean age 23.3 y ±2.66 SE, range 18–26) were recruited**.** All of the participants had normal or corrected-to-normal vision, were naive to the aim of the experiment and presented no neurological or psychiatric history. All provided written informed consent according to institutional guidelines of the local research ethics committee and were paid for their participation. All the participants were debriefed and thanked after their participation.

#### (ii) Stimuli

The recordings of the first 16 B participants were each cut into 45 videos corresponding to the 45 trials performed during the experiment. Videos containing artifacts (e.g., B participants moving beyond of the scope of the webcam, concealing her face with her hand/fingers, or looking away from the screen) were rejected. The resulting stimuli (n = 609) consisted of videos of size 720×421 pixels of length 6 sec projected on a 19-inch black LCD screen and represented 22.5% of the videos recorded during the Experiment 1.

#### (iii) Procedure

The judges were confronted with all the videos, presented in a random order. They were told that they were going to watch videos of women perceiving emotional or non-social movies. Before each trial, a grey screen with the indication “Get ready…” was presented for 400 ms, followed by the video. Participants were asked to press the appropriate key on a keyboard when they recognized joy, fear, or non-social signs in the women facial expressions. They were then required to wait 500 ms for the next video to appear on the screen.

#### (iv) Data analysis

A Cohen-Kappa coefficient test was used to measure the inter-rater agreement. To explore the performance of the judges against chance-level, we performed a series of three-choice binomial tests.

## Results

**Figure 3:**
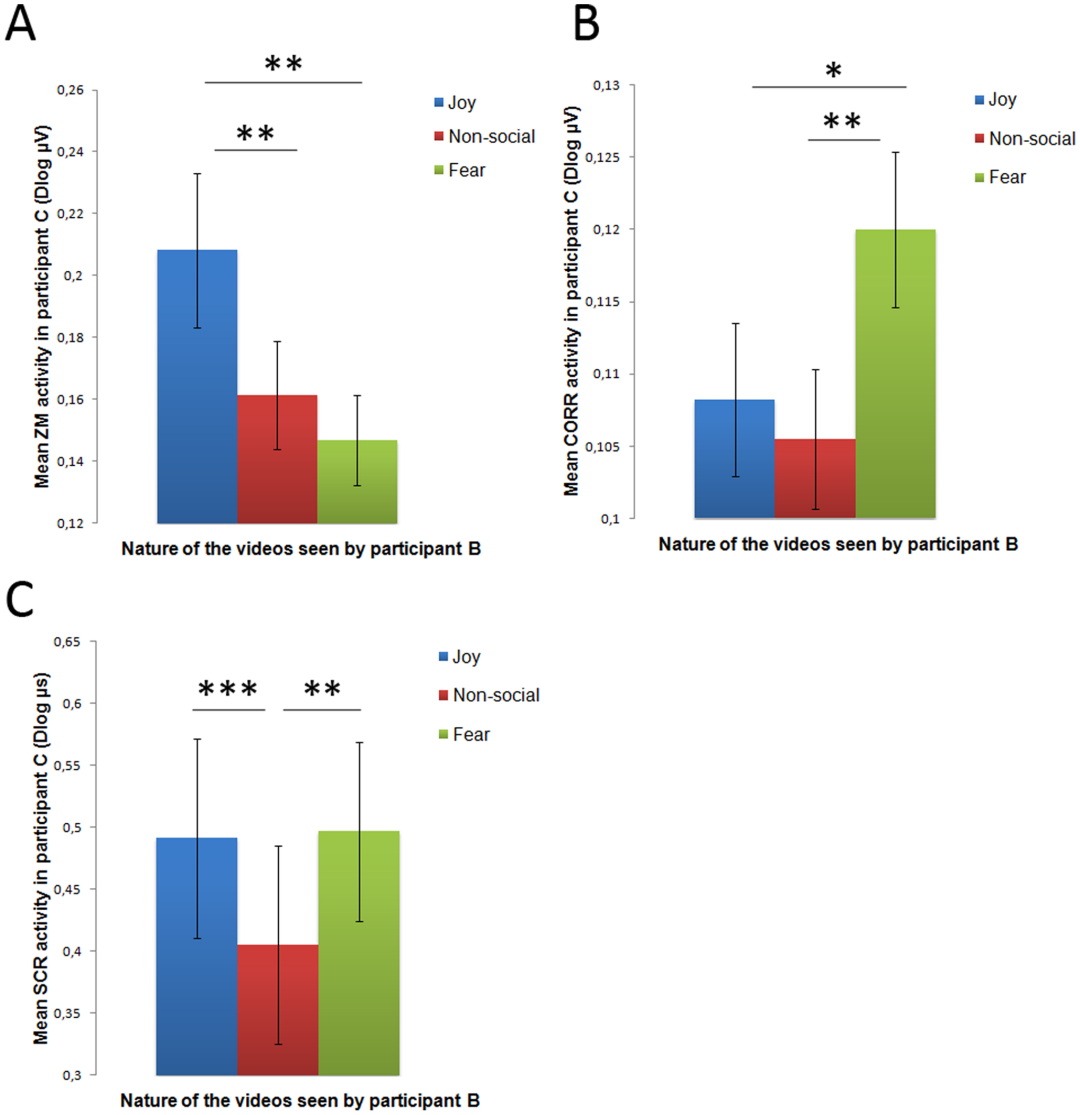
Electromyographic (*zygomaticus major* [ZM] and *corrugator supercilii* [CS]) and skin conductance (SCR) responses in participant C relative to the emotional content perceived by participant B. *(A)* EMG response of ZM in C relative to the emotional content perceived by B. *(B)* EMG response of CS in C relative to the emotional content perceived by B. *(C)* SCR responses in C relative to the emotional content perceived by B. Black lines indicate significant effects at *P<0.05; **P<0.01; ***P<0.001. Error bars indicate SEM.

First, we tested whether facial cues of joy were transitively transmitted from A to C, via B. Figure 3*A* displays the mean ZM response in participants C depending on the emotional content displayed in A and presented to participants B. Typically involved in the production of smiles and preferentially activated during the perception of joy expressions [25], ZM activity was expected to increase in C when B was watching videos depicting joy. Our analysis of ZM activity showed a significant main effect of Emotion (*F*_2, 22_ = 7.96, *p* = 0.001, *ε* = 0.70, *corrected p* = 0.004, β = 0.715, *η^2^* = 0.266). ZM activity was significantly enhanced in C when B was watching joy expressions compared to non-social stimuli (*t*_22_ = 2.90, *p*<0.01, *d* = 0.45) and when compared to fearful expressions (*t*_22_ = 3.05, *p*<0.01, *d* = 0.62). Moreover, ZM activity was not different between fear and non-social conditions (*t*_22_ = 1.39, *p*>0.1). These results show that muscular activity in C was specific of the emotional content observed by B, revealing a transitive motor transmission of joy expressions.

Second, we tested whether facial cues of fear were transitively transmitted. We therefore compared the activity of the CS across the conditions. The CS pulls the brows together and is often used as a measure of negative emotional reactions to negative stimuli [26], notably fear-related stimuli (e.g., snakes in [27] or facial and bodily expressions of fear [14]). Our analysis showed a significant main effect of Emotion (*F*_2, 16_ = 5.46, *p*<0.01, β = 0.752, *η^2^* = 0.334). CS activity was significantly enhanced in C when B was watching fearful expressions compared to non-social stimuli (*t*_16_ = 2.91, *p* = 0.01, *d* = 0.68) and to joy expressions (*t*_16_ = 2.83, *p*<0.05, *d* = 0.53). Last, CS activity was not different between joy and non-social conditions (*t*_16_ = 0.56, *p*>0.1). Figure 3*B* shows the mean CS activity across the conditions. Again, we found that muscular activity in C matched the emotional expressions watched by B.

Third, we tested whether the transmission process also involved an arousal component or whether it was only limited to facial reactivity by comparing the SCR activity across the conditions. The statistical analysis revealed a significant main effect of Emotion (*F*_2, 18_ = 7.32, *p*<0.01, β = 0.924, *η^2^* = 0.289). A significant increase of SCR was found in C when B was watching joy expressions, compared to when she was watching non-social stimuli (*t*_18_ = 3.75, *p* = 0.001, *d* = 0.24). A similar pattern was observed for fear vs. non-social (*t*_18_ = −3.21, *p* = 0.005, *d* = 0.27). Lastly, no difference was found between joy and fear (*t*_18_ = −0.19, *p*>0.1). The results suggest an increase of physiological arousal in C when B was watching emotional content, irrespective of the exact nature of the perceived emotion. Figure 3*C* displays the mean SCR across the conditions.

Finally, to investigate the nature and reliability of information which was transmitted from B to C, three judges who were blind to our hypotheses were requested to explicitly recognize signs of joy, fear or neutrality (when watching non-social cues) on B’s faces, using a forced-choice task, in a follow-up experiment. We performed a Cohen-Kappa coefficient test that provides a measure of inter-rater agreement for qualitative items [28]. This test revealed a strong agreement between the judges (mean *k* value = 0.78; *k* value for joy items = 0.77; *k* value for non-social items = 0.89; *k* value for fear items = 0.67). The judges were at chance-level in recognizing joy signs in B's faces (Three-choice binomial, *p*>.1) and above chance-level in recognizing fear signs and neutrality displayed by B (Three-choice binomial, *p* = 0.01 and *p*<0.001 respectively). These results suggest that the transmission of an emotion from B to C may be independent of an explicit recognition of the emotional signs displayed on B's faces, at least in the situation where B herself perceived joy expressions. Yet, there was a difference between the two experiments: while judges were exposed to several participants B, C was only exposed to two participants. As a consequence, we cannot exclude the possibility that there were differences in susceptibility to emotional cues in B between participants C and judges.

## Discussion

Our findings indicate that emotional expressions of joy and fear can be spontaneously transmitted beyond dyads. Overt expressions of an emotion in an individual A caused in an observer B the involuntary production of subtle cues that induced an emotional reaction in a third individual C (who had perceptual access to B but not to A).

The facial reactions triggered in C were characteristic of the type of emotions displayed by A, as revealed by the EMG responses of our participants. Activity of the ZM muscle region was heightened in C when B perceived the display of joy in A (in the form of facial, bodily and vocal signals) compared to when B was perceiving displays of fear in A or non-social stimuli. Activity of the CS muscle region, on the other hand, was heightened in C when B was observing expressions of fear compared to when B was watching expressions of joy in A or non-social stimuli. Although the use of CS as an index of fearful facial reactions is a limitation to demonstrate a transitive motor transmission of fearful expressions, according to the FACS nomenclature [13], facial expressions of fear usually involve the widening of the eyes (AU5), a raising of the eyebrows (AU1+2: activity of the frontalis) co-occurring with frowning (AU4: activity of CS), as well as the stretching of the mouth sideways (AU20). Thus, if the frontalis activity is indeed used in the EMG literature to measure facial reactions associated with the experience of fear (e.g., [16], [19], [29]), CS is also relevant (e.g., [14], [27]) as it is known to reflect a more general negative facial response and is recruited in fearful facial expressions.

Numerous studies report the production of subtle and specific facial reactions in front of facial, bodily, as well as vocal expressions of emotions [16], [19], [20], [25], [26], [30]–[34]. Here we extend these results to a minimal element of a crowd situation by showing, for the first time, that the perception of the facial reactions of an individual (B), herself perceiving an emotional display (A), triggers the release of a specific facial pattern in a third party (C). Importantly, C’s reactions were not limited to a set of facial motor responses but involved emotional arousal, as evidenced by the increase in SCR during the emotional conditions (joy and fear) compared to the non-social condition. Importantly, the lower SCRs we observed during the non-social condition provide evidence against interpreting SCR increases for fear and joy expressions as the physiological consequences of attentional process only [35]. This increase of SCR response to emotional conditions as compared to non-social condition does not merely reflect an overall increase of arousal for vision of a body versus vision of a non-body stimulus as SCR was recorded in C who only sees a social agent B. Moreover, given that SCR activity is found to be coupled with specific muscular activity during emotional conditions, it is unlikely that observed SCR would not reflect the processing of emotional content.

Of interest here, judges in the follow-up experiment were at chance level when asked to recognize joy cues in B's faces. This indicates that transitive emotional transmission could occur, even in the absence of explicit recognition of emotional information in the pivot individual's face, on the basis of mere unintentional cues. Though it is tempting to generalize this finding to all type of emotions, the fact that cues related to the experience of fear could be recognized in B’s face may also indicate that the extent to which emotional expressions are spontaneously expressed by their observers might well depend on their reference or content. In this respect, expressions related to immediate and urgent threats (such as expressions of fear) might more easily induce explicit cues in the face of their observers. Be that as it may, they must be such unintentional cues that they explain the well-documented fact that emotional contagion can occur without conscious access [8]. An impressive study by Tamietto et al. [14], in particular, reported emotional transmission in cortically-blind patients. Note that one limitation of this study is the absence of physiological measures in B. Further experiments could test each step in the spread of emotions in transitive situations and provide information about a potential decrease in physiological responses from A to C, or conversely, a gradual increase in emotional information, that is to be expected in crowd contexts [4].

Finally, our findings point out an important theoretical issue, the distinction between *cues* and *signals*. Cues can be defined as stimuli that elicit a specific cognitive or behavioral response that goes beyond the mere perception of the cue itself. Signals can be defined as cues that have the *function* of eliciting such a response [36], [37]. Are the subtle emotional cues produced by B and picked up by C a mere side effect of B’s emotional arousal caused by the recognition of A’s emotion, or do these cues have the function of eliciting a similar emotional response in the third party? In other terms, are they not merely cues but signals?

In our study, participant B did not know that she was being observed and did not therefore intend to communicate anything by means of her facial expression (of which she may well have been unaware). The fact that, at least in the case of joy, these expressions were not recognized by judges strongly suggests that participant C’s use of these cues was not intentional either. The cues we are talking about are neither intentionally emitted not intentionally attended to.

The fact that B nevertheless produced unintentional cues strong enough for them to influence participant C can be interpreted as evidence that these emotional cues are biological adaptations, the function of which is to transmit an emotion in a non-intentional way. If so, how is this function adaptive? A possibility worth exploring is that facial activity in B is an evolved cooperative behavior that consists in the unconscious and spontaneous signaling of survival-value information that may induce appropriate emotional and preparatory behavior in our conspecifics. Such a mechanism would be adaptive, on the one hand, in threatening situations where flight and mobbing behaviors are optimal strategies; and, on the other hand, in favorable situations where signaling to conspecifics the presence of non-scarce rewarding features of the environment may foster social bonds. More work would be required to ascertain whether unintended and not consciously attended cues of specific emotion are in fact evolved signals that contribute to the fitness of those who produce them and to that of those who are influenced by them.

Our study, we hope, offers some new insights and raises new questions about the spread of emotions across individuals in group settings. This should help revive a once prolific intellectual tradition – the social psychology of crowds – which has contributed so much to the study of human collective behavior in the past.
